# Nonlinear Adaptive Optimal Controller Design for Anti-Angiogenic Tumor Treatment

**DOI:** 10.3390/biomedicines11020497

**Published:** 2023-02-08

**Authors:** Nitendra Nath, Irfan Kil, Ugur Hasirci, Richard E. Groff, Timothy C. Burg

**Affiliations:** 1 Ford Motor Company, Dearborn, MI 48126, USA; 2 Applied Medical Resources Corporation, Rancho Santa Margarita, CA 92688, USA; 3 Department of Electrical and Electronics Engineering, Duzce University, 81620 Duzce, Turkey; 4 Department of Electrical and Computer Engineering, Clemson University, Clemson, SC 29634, USA; 5 Department of Veterinary Biosciences and Diagnostic Imaging, University of Georgia, Athens, GA 30602, USA

**Keywords:** angiogenesis, anti-angiogenic treatment, control systems, optimal control

## Abstract

Angiogenesis is an important process in tumor growth as it represents the regime when the tumor recruits blood vessels from the surrounding tissue to support further tumor growth. Anti-angiogenic treatments aim to shrink the tumor by interrupting the vascularization of the tumor; however, the anti-angiogenic agents are costly and the tumor response to these agents is nonlinear. Simple dosing schemes, e.g., a constant dose, may yield higher cost or lower efficacy than an approach that considers the tumor system dynamics. Hence, in this study, the administration of anti-angiogenic treatment is considered as a nonlinear control problem. The main aim of the controller design is to optimize the anti-angiogenic tumor therapy, specifically, to minimize the tumor volume and drug dose. Toward this aim, two nonlinear optimal controllers are presented. The first controller ensures exponential tracking of a desired, optimal tumor volume profile under the assumption that all parameters in the system model are known. The second controller, on the other hand, assumes all the parameters are unknown and provides asymptotic tracking. Both controllers take pharmacokinetics and pharmacodynamics into account, as well as the carrying capacity of the vascular network. Lyapunov based arguments are used to design the controllers, using stability arguments, and numerical simulation results are presented to demonstrate the effectiveness of the proposed method.

## 1. Introduction

**Figure 1:**
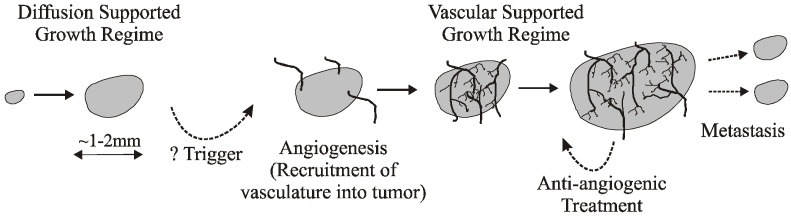
Angiogenesis occurs as the tumor approaches a certain size that can no longer be supported by diffusion alone; the tumor must recruit blood vessels from the surrounding tissue to support further growth.

Breast cancer has the highest incidence rate compared to other types of cancer [1]. An overview of the tumor angiogenesis is provided by the US National Cancer Institute [2] and summarized in Figure 1. Initially, tumor cells get nutrients from the surrounding tissues via diffusion. The diffusion supported growth regime ends when physical limits (1–2 mm of tumor diameter) prevent nutrients and oxygen from reaching the core of the tumor. Then, an angiogenesis triggering event occurs; the tumor cells release factors, such as vascular endothelial growth factor (VEGF) or basic fibroblast growth factor (bFGF) [3], that instigate the recruitment of blood vessels to support further tumor growth. The tumor and support vasculature can then co-develop as the blood vessels supply nutrients and remove waste products from the proliferating tumor cells, and the growing tumor signals additional vascular growth, primarily vascular endothelial cells. The next major event is metastasis when the tumor spreads to the other tissues and organs.

Anti-angiogenic treatment was proposed by Folkman [4] and aimed to control the growth of the vascular network, described above, to indirectly control the tumor. The relationship between the growth of the vascular network and the growth of the tumor is dynamic, complex, and nonlinear. Therefore, applying a fixed or a time-varying dose of anti-angiogenic agents, without observing the response of the tumor, i.e., open-loop control, may fail to shrink the tumor. Closed-loop control may be more effective to control the growth of the vascular network, so as to shrink the tumor, by using feedback signals from the co-developing tumor and vascular network. Since the commercially available anti-angiogenic agents, like angiostatin and endostatin, are expensive, an optimal control approach can be employed to keep the tumor size at a certain (desired) value by using an optimum dose of an anti-angiogenic agent. Ledzewicz and her coworkers have proposed many open-loop [5,6] and closed-loop optimal control schemes [7,8]. Swierniak et al. also presented an optimal controller addressing the same problem in [9]. Nath et al. proposed a novel approach that combines closed-loop tracking control with an optimization to minimize the anti-angiogenic agents that needed to move the system to a setpoint (tumor size) [10]. In 2013, Hasirci et al. proposed enhancements to the core tracking controller: a robust controller in [11] and an adaptive controller in [12]. Researchers have used nonlinear adaptive and robust control techniques in a wide range of applications, success in controlling similar dynamical systems [13,14,15,16,17] inspires the current work.

As a general control design methodology, the salient features of the system to be controlled are first approximated with a mathematical model. In biological systems, especially, it is a good and usual practice to make simplifying assumptions to make the mathematical model tractable, while balancing accuracy and resolution. As experience and evidence build, additional complexities can be added to the model. The controllers reviewed above are based on a simplified dynamic model where tumor volume and vascular carrying capacity are the only state variables. This model assumes drug concentration is equal to drug dose, and also assumes that the effect of the anti-angiogenic agents is instantaneous. A more realistic and, as a result, more complex view of the angiogenesis process dictates the incorporation of pharmacokinetics (PK) and pharmacodynamics (PD) into the system model. As clearly stated in [18], pharmacokinetics equations describe the concentration of a drug and pharmacodynamics equations model the effectiveness of drug dose. Incorporating PK/PD equations to the angiogenic tumor growth model begins to add the clinic reality of *drug delivery* to the model.

In this paper, we present a novel approach for real-time dosing of the anti-angiogenic treatment by considering the richer tumor growth model, which incorporates PK/PD. The main aim of the control design is to optimize the anti-angiogenic tumor therapy for minimizing the tumor volume by using an optimal amount of the anti-angiogenic agents. Two different controllers are presented to achieve the control objective. To minimize the tumor volume, we first formulate a performance index to be minimized and then a nonlinear optimal exact model knowledge controller is designed to drive the tumor volume to an optimal desired time-varying trajectory. Then, a more realistic control design approach is also presented for the same control objective; a nonlinear optimal adaptive controller assuming all the system parameters is unknown. For the adaptive controller, a least-squares estimation technique is used to identify the system parameters in the performance index and the desired optimal trajectory is generated by using an optimization algorithm, which seeks the minimum of the performance index. As demonstrated by simulation results, this optimization algorithm successfully finds the optimum values of the carrying capacity of vasculature and the tumor volume. The paper is organized as follows: Section 2 contains discussion about the modeling issues and a definition of the control problem. Section 3 contains the details of the design of an exact model knowledge nonlinear optimal controller, and then an adaptive controller. Performance results of the proposed adaptive scheme are obtained via numerical simulation in Section 4. The last section discusses the results and highlights some conclusions.

## 2. System Model and Control Problem Definition

Early mathematical models for tumor growth comprised one state variable: tumor volume (*p*(*t*)) [19]. The carrying capacity of the vasculature network (*q*(*t*)) concept was proposed to account for co-growth of the tumor and vasculature, i.e., angiogenesis, and then a killing factor was introduced to model anti-angiogenic therapy, which targets endothelial cells instead of the tumor cells [20]. Many mathematical models have been proposed for this two-state system, some of which attempt to fully describe the complexity of biological process [21,22,23,24,25,26,27]. Since these models are not tractable for mathematical analysis [28], Hahnfeldt et al. proposed and biologically validated a simpler two-state model to describe the interaction between the carrying capacity of vasculature and tumor size [29]. A useful modification of this model was presented by Ergun et al. [30], and more recently by d’Onofrio and Gandolfi [31]. Incorporation of PD/PK equations to this modified model has been presented by Ledzewicz et al. [32] and is given by:

(1) $$\begin{array}{l} \dot{p}=\alpha p\left(1-\frac{p}{q}\right)\\ \dot{q}=bq-dp^{2/3}q-Gscq\\ \dot{c}=-mc+hu\;;\;c\left(t_{0}\right)=0 \end{array}$$

where *p*(*t*) is the tumor volume in mm^3^, *q*(*t*) is the carrying capacity of the endothelial cells, also measured in mm^3^, *c*(*t*) is the plasma concentration of the exogenous angiogenic inhibitors, applied as treatment and measured in [conc.], and *α* is the tumor growth parameter. The term *bq* accounts for the proliferation kinetics of the endothelial cells and the term *dp*^2/3^*q* models endogenous inhibition of the tumor. Because the inhibitors release through the surface of the tumor mass, the 2/3 exponent represents the conversion of the tumor volume into a tumor surface area, and the product *p*^2/3^*q* is due to the interactions of endogenous inhibitors with endothelial cells. The parameters *b* and *d* are constants. The constant *G* denotes the anti-angiogenic killing parameter, and *u*(*t*) is the manipulated control input which corresponds to the anti-angiogenic dose. The drug dose *u*(*t*) and concentration of *c*(*t*) of the inhibitors are linked by a first-order, linear, ordinary differential equation where *m* and *h* are constant parameters [32]. The *effect* of the applied drug is proportional to the concentration of the inhibitor, given as *effect* = *sc* where *s* ∈ [0,1]. Note that “[conc.]” is generic concentration and that the units of concentration do not affect the states *q*(*t*) and *p*(*t*) when the parameters *G*, *m*, and *h* are written for a specific unit of concentration.

**Assumption** **1.** Only the biologically realistic domain where all the state variables are always greater than zero for all time instants, i.e., p(t) > 0, q(t) > 0, and c(t) > 0, will be considered. It is assumed that the tumor volume p(t), the carrying capacity of the vascular network q(t), and the concentration of the angiogenic inhibitors c(t) are measurable.

The control objective is to minimize the tumor volume, *p*(*t*), by using an optimum dose of angiogenic inhibitors, *u*(*t*). This objective is met if the following performance index is minimized:

(2) $$J=p+{\left(\frac{Su}{d}\right)}^{3/2}$$

where *S* = *Gs*. The performance index *J*(*t*) ∈ℝ captures the treatment goal by using the summation of tumor size and drug dose. To obtain the same units, mm^3^, for each term in (2), the drug dose is multiplied by a factor of *S*/*d* and then raised to 3/2. The steady-state expression of the performance index is:

(3) $$J_{0}=p_{0}+{\left(\frac{Su_{0}}{d}\right)}^{3/2}$$

where *J*_0_, *p*_0_, and *u*_0_ are the steady-state values of *J*, *p*, and *u*, respectively. The minimum of this performance index gives the minimum tumor volume that can be obtained by using the minimum drug dose; these values are found from the steady-state properties of the system given in (1) by calculating its equilibria corresponding to a constant drug dose, *u*_0_. By setting left-hand sides of the state equations in (1) to zero, an equilibrium at *p*_0_ = *q*_0_ = 0 is obtained, which is not an admissible point [26], and:

(4) $$p_{0}=q_{0}={\left(\frac{b-Sc_{0}}{d}\right)}^{3/2},\;c_{0}=\frac{h}{m}u_{0}.$$

**Figure 2:**
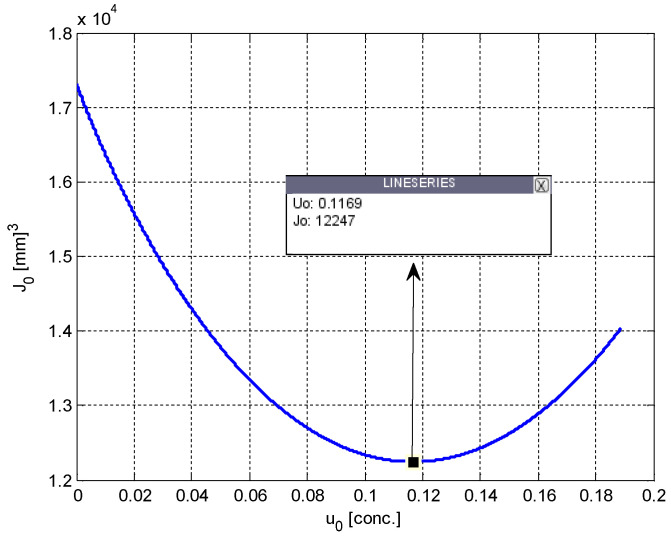
Variation of the steady-state performance index, *J*_0_, with respect to the steady-state drug dose, *u*_0_.

**Figure 3:**
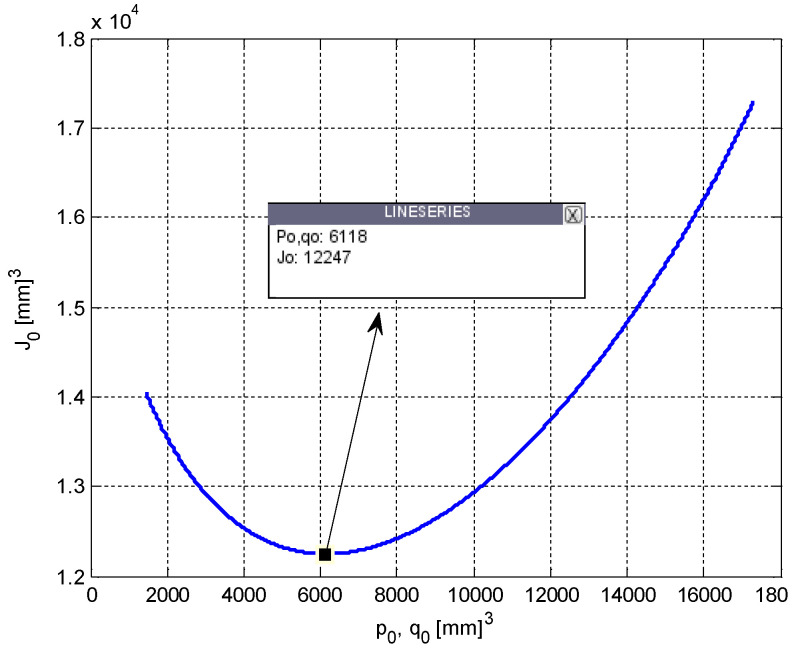
Variation of the steady-state performance index, *J*_0_, with respect to the steady-state values of the tumor volume, *p*_0_, and the carrying capacity, *q*_0_.

This equilibrium point analysis reveals some important properties of the system given in (1). First, it is clear that the tumor volume is equal to the carrying capacity at steady-state. It is also clear that an uninterrupted or a long-term therapy is needed to prevent the growth of the tumor. By selecting the value of the parameter related to PD as *s* = 0.8, and by setting the PK parameters *m* = *h* = 1, which is a case often considered in the literature [33], and finally, by using the system parameter values taken from [31] and given in Table 1, respective equilibria of the tumor volume and the carrying capacity are found as *p*_0_ = *q*_0_ = 17,320 mm^3^, if the control input *u*(*t*) is equal to zero. Figure 2 shows the variation of *J*_0_ with respect to the steady-state values of *u*(*t*), denoted *u*_0_, and Figure 3 shows the variation of *J*_0_ with respect to the steady-state values *p*(*t*) and *q*(*t*), denoted by *p*_0_ and *q*_0_ for selected parameter values.

The minimum of the curves in Figure 2 and Figure 3, i.e., the optimum values of the variables, are found to be:

(5) $$\begin{array}{l} J*=12,247{\;[\mathrm{mm}]}^{3}\\ p*=q*=6118{\;[\mathrm{mm}]}^{3}\\ u*=0.1169\;[\mathrm{conc}.]. \end{array}$$

Considering the results of the analysis given above, the control objective can be more precisely defined as driving *q*(*t*) to its optimum value, q*. Since the tumor volume is equal to the carrying capacity of the vascular network at steady-state, this necessarily means that the tumor volume, *p*(*t*), will also be driven to q* = p* by using the minimum drug dose, if the control objective is achieved.

## 3. Control Design

In this section, two nonlinear optimal backstepping controllers are presented. The first controller encapsulates an exact-model knowledge controller that ensures exponential tracking of a desired optimal tumor volume under the assumption that all parameters in the system model are known. The second controller, on the other hand, is an adaptive controller that supports the practical reality that the parameters are unknown and provides asymptotic tracking.

### 3.1. Exact Model Knowledge Controller

**Figure 4:**
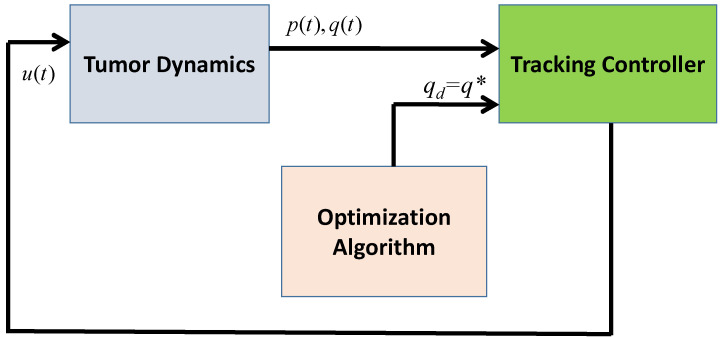
A block diagram representation of the developed tumor minimization technique.

Assuming all parameters in the tumor model are known, a complete control scheme is shown in Figure 3. The tracking controller uses feedback to drive the tumor volume to a desired, optimal point, *q_d_.* That optimal point, *q_d_*, is known a priori from the static optimization summarized in (5). A tracking controller shown in Figure 4 is needed that moves the tumor system towards this optimum value.

To quantify the performance of the exact model tracking knowledge controller to be designed, an error signal can be defined as:

(6) $$e_{1}=q-q*.$$

Differentiating and substituting from (1) yields the error dynamics:

(7) $${\dot{e}}_{1}=bq-dp^{2/3}q-Gscq.$$

This equation lacks a control input to be manipulated to stabilize the error variable; the actual control input appears in the last state equation given in (1). In such cases of coupled subsystems, the backstepping technique [34] provides a tool to design a control input signal that is propagated through the coupled subsystems. To apply this procedure, a virtual control input, *c_d_*(*t*), is added and subtracted to the right-hand side of (7) as:

(8) $${\dot{e}}_{1}=bq-dp^{2/3}q-Scq-Sc_{d}q+Sc_{d}q.$$

In practice, *c_d_*(*t*) is the desired trajectory for the concentration of the inhibitor. A new error variable, *e*_2_(*t*), is defined to quantify the difference between the actual concentration of the inhibitor and the desired concentration of the inhibitor as:

(9) $$e_{2}=c-c_{d}$$

Temporarily ignoring the last two terms in (9), a desired trajectory for the concentration of the inhibitors, that would force *e*_1_(*t*) towards zero, can be designed as:

(10) $$c_{d}=\frac{1}{Sq}\left(bq-dp^{2/3}q+k_{1}e_{1}\right)$$

where *k*_1_ is a positive control gain. Substituting the proposed trajectory yields the closed-loop dynamics for *e*_1_(*t*) as:

(11) $${\dot{e}}_{1}=-k_{1}e_{1}-Sqe_{2}.$$

Investigating the *e*_2_(*t*) dynamics yields:

(12) $$\begin{array}{c} {\dot{e}}_{2}=\dot{c}-{\dot{c}}_{d}\\ \;\;\;\;\;\;\;\;\;\;\;\;\;\;\;=-mc+hu-\frac{d}{dt}\left\{\frac{1}{Sq}\left(bq-dp^{2/3}q+k_{1}e_{1}\right)\right\}\\ \;\;=W\theta +hu \end{array}$$

where

(13) $$W=\left[-c\;\frac{2}{3}p^{2/3}\left(1-\frac{p}{q}\right)\;k_{1}qe_{2}\;k_{1}^{2}e_{1}\right]$$

is a 1-by-4 regression vector containing measurable variables and known gains, and

(14) $$\theta ={\left[\begin{array}{cccc} m & \frac{\alpha d}{S} & S & 1 \end{array}\right]}^{T}$$

is a 4-by-1 vector containing the parameters. The actual control input, the concentration of angiogenic inhibitor, can be designed as:

(15) $$u=\frac{1}{h}\left(-k_{2}e_{2}-W\theta +Sqe_{1}\right)$$

where *k*_2_ is a positive control gain, which renders:

(16) $${\dot{e}}_{2}=-k_{2}e_{2}+Sqe_{1}$$

**Remark** **1.** *The above approach means that q(t) will be controlled directly, and that p(t) is indirectly controlled in the sense that it follows q(t) towards an equilibrium. From (1) and Assumption 1, it is evident that $\dot{p}(t)\leq 0$ when p(t)≥q(t), thus p(t) decreases. If p(t)<q(t), then p(t) will increase until p(t)=q(t). It will start to decrease again if p(t)≥q(t). Therefore, p(t) is bounded as long as q(t) is bounded.*

**Theorem** **1.** For the tumor growth model given in (1), the dose of the angiogenic inhibitor applied according to (15) ensures that the concentration of the inhibitor exponentially follows its trajectory, c_d_(t), in order for the carrying capacity, q(t), of the vascular network to reach the constant value, q*, exponentially fast. In other words, e_1_ goes to zero exponentially fast. It also guarantees that all signals in the closed-loop system are bounded.

**Proof** **1.** Consider the following nonnegative Lyapunov function:

(17) $$V_{1}=\frac{1}{2}e_{1}^{2}+\frac{1}{2}e_{2}^{2}.$$

Taking the time derivative and then substituting the closed-loop dynamics of *e*_1_ and *e*_2_, given by (11) and (16), respectively, yields:

(18) $${\dot{V}}_{1}=-k_{1}e_{1}^{2}-k_{2}e_{2}^{2}.$$

By considering (17) and (18) together, one can write:

(19) $$V_{1}=V_{1}(t_{0})\exp\left\{-\eta t\right\},\eta \in {\mathbb{R}}_{+}$$

where *V*_1_(*t*_0_) is the initial condition of *V*_1_(*t*) and exp{·} is the base of the natural logarithm. This means that *V*_1_(*t*) and its components, *e*_1_(*t*) and *e*_2_(*t*), go to zero exponentially. According to (6), if *e*_1_(*t*) goes to zero, then the carrying capacity, *q*(*t*), is bounded, i.e., $q(t)\in L_{\infty }.$ From Remark 1, $p(t)\in L_{\infty }$; therefore, $c_{d}(t)\in L_{\infty }.$ From (9), it can be concluded, $c(t)\in L_{\infty }.$ Finally, from (15), $u(t)\in L_{\infty }.$ □

**Figure 5:**
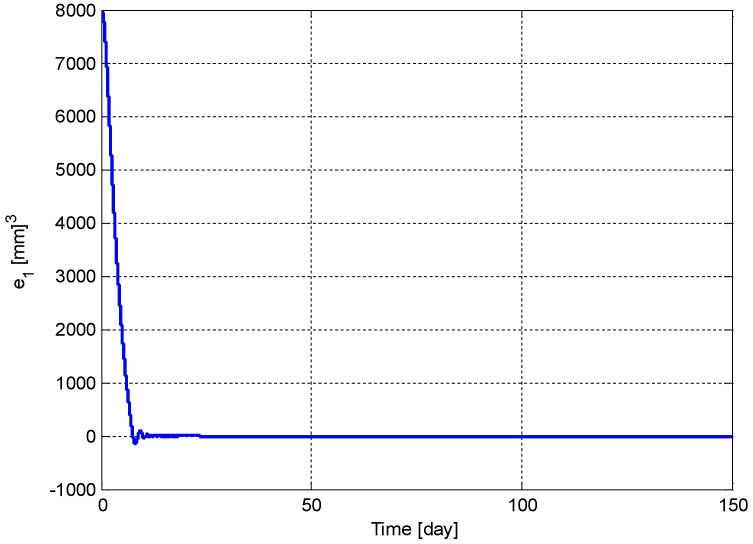
Variation of the tracking error, $e_{1}(t)$.

**Figure 6:**
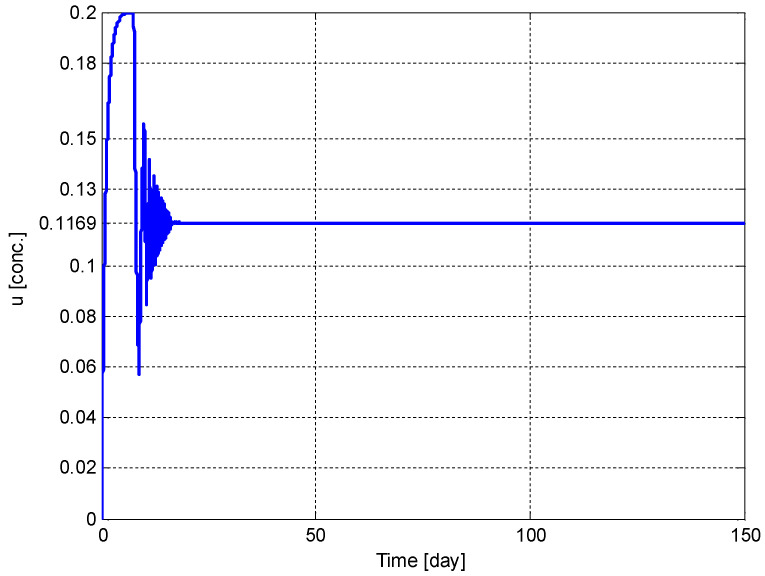
Variation of the dose of the angiogenic inhibitors, *u(t)*.

**Figure 7:**
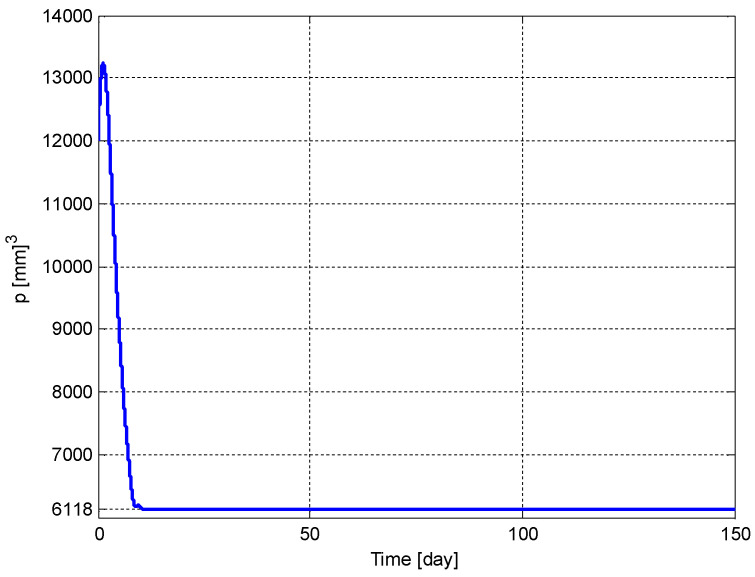
Variation of the tumor volume, *p*(*t*).

The following simulation is presented to illustrate the performance and feasibility of the proposed controller applied to the tumor volume problem defined in this section. Note that the approach used to design this exact model controller will be expanded to account for parameter uncertainty in the following section. The simulation parameter values were provided in Section 2. Figure 5 shows the time variation of the error between the actual and optimum carrying capacity, defined in (6)—it goes to zero exponentially, as claimed. Practically, this means that the actual value of the carrying capacity of the vascular network goes to its optimum value, 6118 mm^3^, if the dose of the angiogenic inhibitors is applied to the tumor, as in Figure 6. This figure also demonstrates that the required dose of the anti-angiogenic agents is bounded. Finally, Figure 7 illustrates that the tumor volume follows the carrying capacity to reach its optimum value at steady-state. Values of the control gains were selected as *k*_1_ = 0.1 and *k*_2_ = 10 for this simulation. Initial values were assigned as *p*(*t*_0_) = 12,000 mm^3^ and *q*(*t*_0_) = 14,000 mm^3^. This approach is now extended to account for parameter uncertainty in the vector in (14).

### 3.2. Adaptive Controller

Determination of the “exact” values of the system parameters *b*, *d*, and *S*, which appear in the carrying capacity dynamics, for a specific patient is a very difficult and time-consuming task. The biological experiments that would be required to measure the exact values of the parameters for each patient would be costly, may delay the start of treatment, and would likely ultimately fail because of the uncertainty in measuring such a complex biological system. A more realistic and clinically feasible approach is to achieve the control objective, to minimize the tumor volume, *p*(*t*), by using an optimum dose of angiogenic inhibitors, *u*(*t*), despite measurement uncertainties in tumor growth model parameters. To end this, an estimate of the performance index given in (2) is defined, denoted by $\hat{J}(t)\in \mathbb{R}$, as:

(20) $$\hat{J}=p+{\left(\frac{\hat{S}c}{\hat{d}}\right)}^{3/2}$$

where $\hat{S}(t),\hat{d}(t)\in \mathbb{R}$ are the estimates of the parameters *S* and *d*, respectively. Such a definition reveals an important issue with the control problem definition; the control problem will no longer be a set point problem, i.e., the desired trajectory for the carrying capacity will not be a fixed point, q*. Instead, the desired carrying capacity will be a time-varying trajectory as knowledge about the model parameters evolves. 

**Figure 8:**
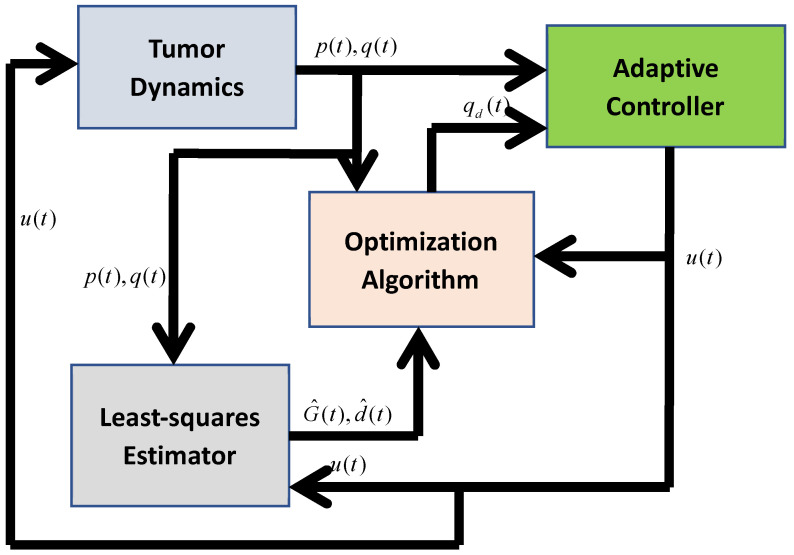
A block diagram representation of the developed adaptive tumor minimization technique.

Figure 8 shows an outline of the solution and indicates the three key components. First, the calculation of the performance index given in (20) requires the individually estimated values of the system parameters; a convergent estimation scheme is proposed to find the estimates of these parameters. Specifically, a least-squares estimation technique is adopted to generate the estimates of the unknown constant parameters. Second, an adaptive controller is proposed so that the carrying capacity of the vascular network follows a time-varying desired optimum trajectory $q_{d}(t)$, that is $q(t)\to q_{d}(t)$ as t→∞. Third, the desired trajectory, $q_{d}(t)$, is generated dynamically and online, using a numerical optimization method to minimize the performance index given by (20), such that $q_{d}(t)\to q*$ where q* is the optimum value of q(t) at steady-state.

#### 3.2.1. Least-Squares Parameter Estimator

The least-squares estimation technique is used in many types of parameter estimation schemes. The main idea behind the technique is to extract the maximum information about the unknown parameters by using the measurable quantities in the system. Since the tumor volume, carrying capacity, and the concentration of the inhibitors are assumed to be measurable, information about the unknown parameters can be extracted as explained in the following.

To facilitate the estimator development, the *q*-dynamics given in (1) are parameterized as:

(21) $$Q=W_{e}\theta_{e}$$

where *Q* denotes $\dot{q}(t)$,

(22) $$W_{e}(t)≜\left[q\;-p^{2/3}q\;-cq\right]\in {\mathbb{R}}^{1\times 3}$$

is a measurable regression vector, and

(23) $$\theta_{e}={\left[\begin{array}{ccc} b & d & S \end{array}\right]}^{T}\in {\mathbb{R}}^{3}$$

is a vector of unknown parameters. Such a parameterization allows the design of an adaptive estimation rule for the unknown parameters, given in (23), through the known quantities found in (22). To further facilitate the estimator design, a prediction error ε(t)∈ℝ is defined as:

(24) $$\varepsilon ≜Q_{f}-{\hat{Q}}_{f}$$

in which $Q_{f}(t)\in \mathbb{R}$ is a filtered signal defined in the form of:

(25) $${\dot{Q}}_{f}≜-\beta Q_{f}+\beta Q\;;\;Q_{f}(t_{0})=0$$

where β∈ℝ is a positive constant. It should be noted that (25) cannot be implemented directly since *Q*(*t*) is not measurable; however, an implementable form of this signal is provided in Appendix A. ${\hat{Q}}_{f}(t)\in \mathbb{R}$ is the estimate of *Q_f_*(*t*) and is defined as:

(26) $${\hat{Q}}_{f}≜W_{f}{\hat{\theta }}_{e}$$

where $W_{f}(t)\in {\mathbb{R}}^{1\times 3}$ is a filtered regression vector expressed as:

(27) $${\dot{W}}_{f}≜-\beta W_{f}+\beta W_{e}\;;\;W_{f}\left(t_{0}\right)=0_{1\times 3}$$

and 0_1×3_ denotes a 1-by-3 vector of zeros. In (26), ${\hat{\theta }}_{e}={\left[\begin{array}{ccc} \hat{b} & \hat{d} & \hat{S} \end{array}\right]}^{T}\in {\mathbb{R}}^{3}$ is the estimate vector of unknown parameters. Substituting (21) into (25) yields:

(28) $${\dot{Q}}_{f}+\beta Q_{f}=\beta W_{e}\theta_{e}.$$

The last expression can be rewritten as:

(29) $${\dot{Q}}_{f}+\beta Q_{f}={\dot{W}}_{f}\theta_{e}+\beta W_{f}\theta_{e}$$

where (27) was used. After taking the time derivative of (26), and then adding and subtracting the term ${\dot{W}}_{f}{\hat{\theta }}_{e}$ to the right-hand side of the resulted expression, the following expression will be obtained:

(30) $${\dot{\hat{Q}}}_{f}+\beta {\hat{Q}}_{f}=\frac{d}{dt}\left(W_{f}{\hat{\theta }}_{e}\right)+\beta W_{f}{\hat{\theta }}_{e}$$

where (26) and (27) were used. After subtracting (30) from (29), and utilizing (24) and (27), the resulting expression can be written as:

(31) $$\dot{\varepsilon }+\beta \varepsilon =\frac{d}{dt}\left(W_{f}{\tilde{\theta }}_{e}\right)+\beta W_{f}{\tilde{\theta }}_{e}$$

where ${\tilde{\theta }}_{e}\in {\mathbb{R}}^{3}$ is the estimation error signal, defined as:

(32) $${\tilde{\theta }}_{e}≜\theta_{e}-{\hat{\theta }}_{e}.$$

From (31), it can be shown that a mathematically useful, but unrealizable form of the prediction error ε(t) given in (24) can be written as [35]:

(33) $$\varepsilon =W_{f}{\tilde{\theta }}_{e}.$$

Based on the subsequent analysis, the following continuous least-squares update law ${\dot{\hat{\theta }}}_{e}\in {\mathbb{R}}^{3}$ is employed for estimating the unknown parameters:

(34) $${\dot{\hat{\theta }}}_{e}≜\Gamma W_{f}^{T}\varepsilon$$

where $\Gamma (t)\in {\mathbb{R}}^{3\times 3}$ is the least-squares estimation gain matrix which is designed as:

(35) $${\dot{\Gamma }}^{-1}≜W_{f}^{T}W_{f}.$$

**Remark** **2.** *If Q(t) is bounded, from (25), it can be seen that*$Q_{f}(t),{\dot{Q}}_{f}(t)\in L_{\infty }.$*Similarly, if*$W_{e}(t)\in L_{\infty },$*is bounded, from (27), it can be seen that*$W_{f}(t),{\dot{W}}_{f}(t)\in L_{\infty }$ [35].

**Remark** **3.** *It should be noted that if*$\Gamma^{-1}\left(t_{0}\right)$*is selected to be positive definite and symmetric, then*$\Gamma \left(t_{0}\right)$*is also positive definite and symmetric. Therefore, it follows that both*$\Gamma^{-1}\left(t\right)$*and*$\Gamma \left(t\right)$*are positive definite and symmetric. The following expression can be obtained from (35):*

(36) $$\dot{\Gamma }=-\Gamma W_{f}^{T}W_{f}\Gamma .$$

*It can be easily seen from (36) that*$\dot{\Gamma }\left(t\right)$*is negative semidefinite; therefore,*$\Gamma \left(t\right)$*is always constant or decreasing. Hence, it follows that*$\Gamma \left(t\right)$*is bounded* [34,36].

**Theorem** **2.** *The update law defined in (34) ensures that*$\left\|{\tilde{\theta }}_{e}\right\|\to 0$*as*t→∞*, provided that the following Persistency of Excitation condition* [36] *holds:*

(37) $$\kappa_{1}I_{3}\leq \int_{t_{0}}^{t_{o}+\delta }W_{f}^{T}\left(\tau \right)W_{f}\left(\tau \right)d\tau \leq \kappa_{2}I_{3}$$

*where* $\kappa_{1},\kappa_{2},\delta \in \mathbb{R}$ *are positive constants and I_3_ is a standard 3-by-3 identity matrix.*

**Proof** **2.** See Appendix B. □

#### 3.2.2. Adaptive Controller Design

The parameter estimates, ${\hat{\theta }}_{e}(t)$, are ultimately used to generate the desired trajectories; that is, the tracking objective for the control system. The current challenge is to now create a tracking controller for a system with uncertain parameters—this suggests an adaptive controller solution. To proceed with the development of an adaptive control law to achieve the control objective, we again consider *c*(*t*) as the virtual control input and define the error variables to rewrite the error dynamics as:

(38) $$e_{a1}=q-q_{d}$$

(39) $$e_{a2}=c-c_{d}.$$

As stated before, *c_d_*(*t*) is the desired optimal trajectory for the concentration of the inhibitors. Note that *q*-dynamics can be rewritten as:

(40) $$\begin{array}{cc} A_{1}\dot{q} & =A_{1}bq-A_{1}dp^{2/3}q-cq\\ & =W_{1}\theta_{1}-cq \end{array}$$

where $A_{1}≜1/S,$ $W_{1}=\left[\begin{array}{cc} q & -p^{2/3}q \end{array}\right]\in {\mathbb{R}}^{1\times 2}$ is a 1-by-2 regression vector containing measurable variables and $\theta_{1}={\left[\begin{array}{cc} A_{1}b & A_{1}d \end{array}\right]}^{T}\in {\mathbb{R}}^{2}$ is a 2-by-1 vector containing the uncertain parameters. After substituting (39) into (40), the following expression is obtained:

(41) $$A_{1}\dot{q}=W_{1}\theta_{1}-e_{a2}q-c_{d}q.$$

After taking the time-derivative of (38) and multiplying both sides of the resulting expression by *A*_1_, the following expression will be obtained:

(42) $$A_{1}{\dot{e}}_{a1}=W_{2}\theta_{2}-e_{a2}q-c_{d}q$$

where (41) was utilized. In (42), $W_{2}=\left[\begin{array}{cc} W_{1} & -{\dot{q}}_{d} \end{array}\right]\in {\mathbb{R}}^{1\times 3}$ is a 1-by-3 regression vector containing measurable variables and $\theta_{2}={\left[\begin{array}{cc} \theta_{1}^{T} & A_{1} \end{array}\right]}^{T}\in {\mathbb{R}}^{3}$ is a 3-by-1 vector containing the uncertain parameters. Then, the desired trajectory for the concentration of the inhibitors can be designed as:

(43) $$c_{d}=\frac{1}{q}\left(W_{2}{\hat{\theta }}_{2}+k_{a1}e_{a1}\right)$$

in which *k_a_*_1_ is a positive control gain. In (43), ${\hat{\theta }}_{2}(t)\in {\mathbb{R}}^{3}$ is an estimate of the constant vector, $\theta_{2}.$ A parameter estimation error vector can be defined to quantify estimation performance as:

(44) $${\tilde{\theta }}_{2}=\theta_{2}-{\hat{\theta }}_{2}.$$

Then the final dynamics for *e_a1_* will be:

(45) $$A_{1}{\dot{e}}_{a1}=-k_{a1}e_{a1}-e_{a2}q+W_{2}{\tilde{\theta }}_{2}.$$

Following the same procedure, i.e., dividing both sides of the *c*-dynamics given in (1) by *h* and then substituting the results into the time derivative of (39), one can write:

(46) $$A_{2}{\dot{e}}_{a2}=W_{3}\theta_{3}+u$$

where $A_{2}≜h^{-1}\in \mathbb{R},$ $W_{3}=\left[\begin{array}{cc} -c & -{\dot{c}}_{d} \end{array}\right]\in {\mathbb{R}}^{1\times 2}$ is a 1-by-2 regression vector containing measurable variables and $\theta_{3}={\left[\begin{array}{cc} mA_{2} & A_{2} \end{array}\right]}^{T}\in {\mathbb{R}}^{2}$ is a 2-by-1 vector containing the uncertain parameters. Based on the subsequent stability analysis and the control input, the concentration of angiogenic inhibitors, can be designed as:

(47) $$u=-W_{3}{\hat{\theta }}_{3}-k_{a2}e_{a2}+e_{a1}q$$

where *k_a_*_2_ is a positive control gain and ${\hat{\theta }}_{3}(t)\in {\mathbb{R}}^{2}$ is an estimate of the constant vector $\theta_{3},$ which renders:

(48) $$A_{2}{\dot{e}}_{a2}=-k_{a2}e_{a2}+qe_{a1}+W_{3}{\tilde{\theta }}_{3}$$

where

(49) $${\tilde{\theta }}_{3}=\theta_{3}-{\hat{\theta }}_{3}.$$

**Theorem** **3.** *For the tumor growth model given in (1), the concentration of the angiogenic inhibitors proposed in (47) ensures that the carrying capacity of the vascular network follows a desired trajectory, q_d_(t), despite uncertainty about the values of parameters in the tumor dynamics with update laws:*

(50) $$\begin{array}{l} {\dot{\hat{\theta }}}_{2}≜\gamma_{1}W_{2}^{T}e_{a1}\\ {\dot{\hat{\theta }}}_{3}≜\gamma_{2}W_{3}^{T}e_{a2} \end{array}$$

*where* $\gamma_{1},\gamma_{2}\in \mathbb{R}$ *are positive constants. In other words, e_a1_ and e_a2_ go to zero. It also guarantees that all signals in the closed-loop system are bounded.*

**Proof** **3.** See Appendix C. □

#### 3.2.3. Optimum Trajectory Generation

The desired carrying capacity input to the adaptive controller will be a time-varying trajectory due to lack of knowledge about the model parameters, i.e., the cost function is changed based on parameter updates from the least-squares estimator. The gradient descent algorithm, also known as steepest descent, is a first-order optimization algorithm to minimize a function. For a function defined by a set of parameters, the gradient descent algorithm starts with an initial set of parameter values and then, by taking steps proportional to the negative of the gradient of the function, reaches the set of parameter values that minimizes the function. In general, it is considered as one of the simplest optimization algorithms. It is used in this overall approach to determine an optimum trajectory for the carrying capacity.

The desired trajectory, *q_d_*(*t*), is needed as the input to the adaptive controller. This continuous optimum trajectory is designed to minimize the performance index given in (20), where the estimates of parameters *S* and *d*, obtained as described in Section 3.2.1, are utilized. For the minimization of the estimate of the performance index, a gradient descent algorithm is employed, which guesses the optimum value ${\bar{q}}_{d}[n]$ at each time step of the algorithm [10]. The output of the algorithm, ${\bar{q}}_{d}[n]$, is passed through a second-order, stable, and proper low-pass filter to generate the continuous and bounded signals $q_{d}(t)$ and ${\dot{q}}_{d}(t).$ The following filters are utilized:

(51) $$q_{d}(t)=\frac{\varsigma_{1}}{\varsigma_{2}s^{2}+\varsigma_{3}s+\varsigma_{4}}{\bar{q}}_{d}[n]$$

(52) $${\dot{q}}_{d}(t)=\frac{s\varsigma_{1}}{\varsigma_{2}s^{2}+\varsigma_{3}s+\varsigma_{4}}{\bar{q}}_{d}[n]$$

where $\varsigma_{1},\;\varsigma_{2},\;\varsigma_{3},\;\varsigma_{4}\in \mathbb{R}$ are positive filter constants and n∈ℤ is a positive integer that represents the iteration step of the algorithm. At step *n*, the optimum trajectory holds the output, ${\bar{q}}_{d}[n]$, constant until the response of the closed-loop system, q(t), has reached a steady-state near $q_{d}(t).$ A new target optimum, ${\bar{q}}_{d}[n+1]$, is then issued. In other words, the algorithm waits for certain thresholds to be satisfied before it proceeds to the next iteration. For instance, if $\left|q(t)-q_{d}(t)\right|\leq {\bar{e}}_{1},$ $\left|{\bar{q}}_{d}[n]-q_{d}(t)\right|\leq {\bar{e}}_{2},$ and $\left|q(t)-p(t)\right|\leq {\bar{e}}_{3},$ then *n* = *n* + 1, where ${\bar{e}}_{1},\;{\bar{e}}_{2},\;{\bar{e}}_{3}\in \mathbb{R}$ are threshold constants. Furthermore, the desired trajectory can be concluded to have converged when the gradient of $\hat{J}(t)$, with respect to q(t), is within a certain threshold. Once $q_{d}(t)$ has reached the termination threshold, the optimization algorithm stops updating ${\bar{q}}_{d}[n].$ As the performance index approaches its minimum value, the desired trajectory $q_{d}(t)$ and u(t) approach $\hat{q}*$ and u*, respectively. $\hat{q}*,\;u*\in \mathbb{R}$ are the estimates of the optimum values of q(t) and u(t), respectively, that result from the optimum seeking algorithm. As mentioned earlier, at steady-state p(t)=q(t); therefore, $p(t)\to p_{d}(t)\to p*$ as $q(t)\to q_{d}(t)\to q*$, where $p_{d}(t)=q_{d}(t)$ and $\hat{p}*=\hat{q}*.$ Then, $\hat{p}*$ is the estimated optimum tumor volume that can be realized by applying the estimated optimum drug dose, $\hat{u}*.$

## 4. Simulation Results

A numerical simulation study was conducted to evaluate the proposed tumor treatment optimization technique using the MATLAB/Simulink environment. It should be noted that the least-squares estimator developed in Section 3.2.1, adaptive controller designed in Section 3.2.2, and the optimization algorithm described in Section 3.2.3 were run simultaneously, as shown in Figure 8. The parameter values were taken from [26] and are summarized in Table 1. Initial values were selected as *p*(*t*_0_) = 10,000 mm^3^ and *q*(*t*_0_) = 12,000 mm^3^ so that $q\left(t_{0}\right)>p\left(t_{0}\right),$ i.e., the carrying capacity of the endothelial cells is greater than the tumor volume; thus, the tumor volume is susceptible to an increase in the absence of a proper therapy.

**Figure 9:**
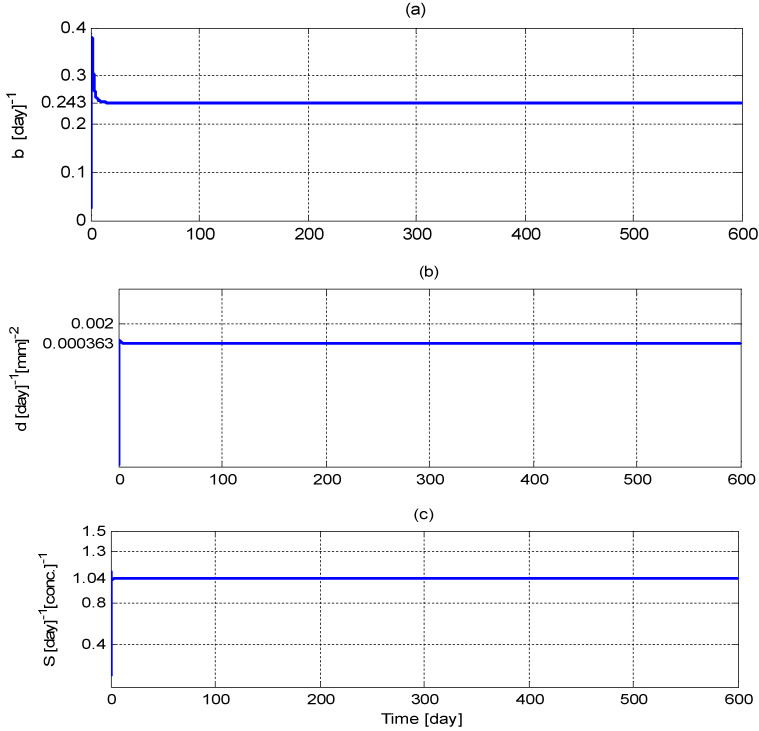
Least-squares estimation: (**a**) $\hat{b}(t)$, (**b**) $\hat{d}(t)$, and (**c**) $\hat{S}(t)$.

The least-square estimator given in (34) was initialized as ${\hat{\theta }}_{e}\left(t_{0}\right)=0.1\theta_{e}$ and the estimation gain matrix was initialized as $\Gamma^{-1}\left(t_{0}\right)=10I_{3}.$ The positive constant β introduced in (25) was set to β=0.05. The adaptive update law was initialized as ${\hat{\theta }}_{a}\left(t_{0}\right)=0_{3}$, where 0_3_ denotes a 3-by-1 vector of zeros. The positive control gains were set to $k_{a1}=10,\;k_{a2}=5,$ and $\gamma_{1}=\gamma_{2}=1\times 10^{-12}.$ The initial guess, ${\bar{q}}_{d}[n],$ was selected to be 7500 mm^3^. Figure 9 shows the least-squares estimate of the unknown vector, $\theta_{e}$; it can be seen from this figure that all the parameters are accurately identified.

**Figure 10:**
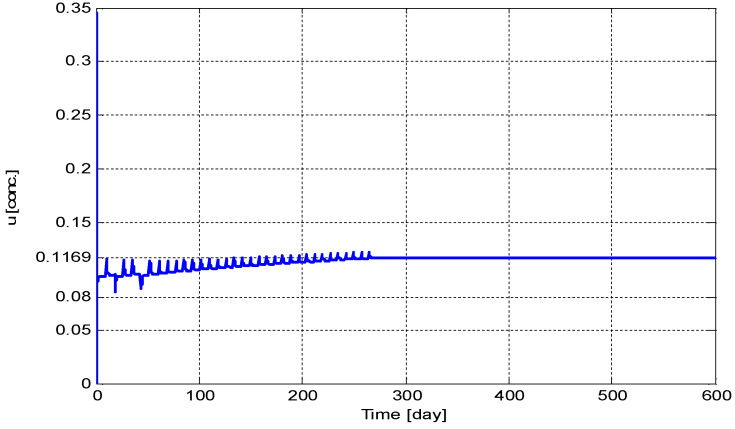
The time evolution of the drug dose, *u*(*t*).

Figure 10 shows the time evolution of the drug dose, u(t). It can be seen from Figure 10 that u(t) converges to its optimum value u*=0.1169 [conc.].

**Figure 11:**
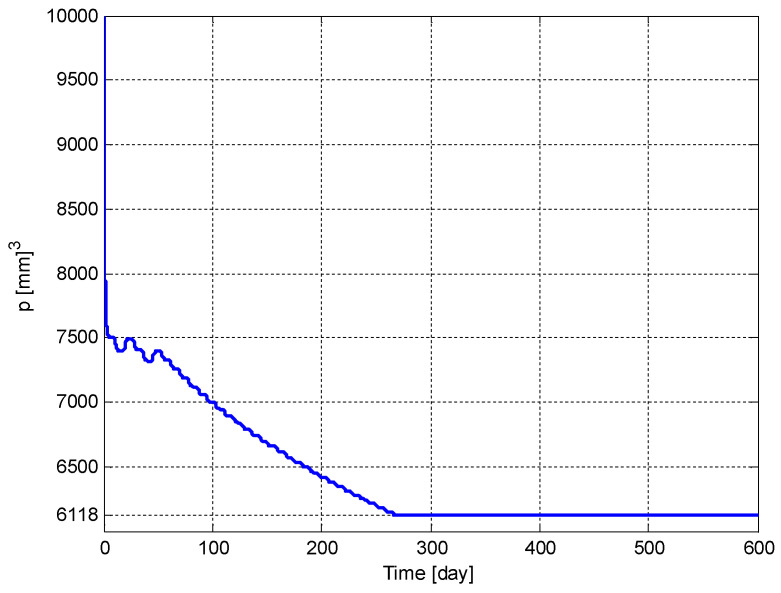
The time evolution of the tumor volume, *p*(*t*).

**Figure 12:**
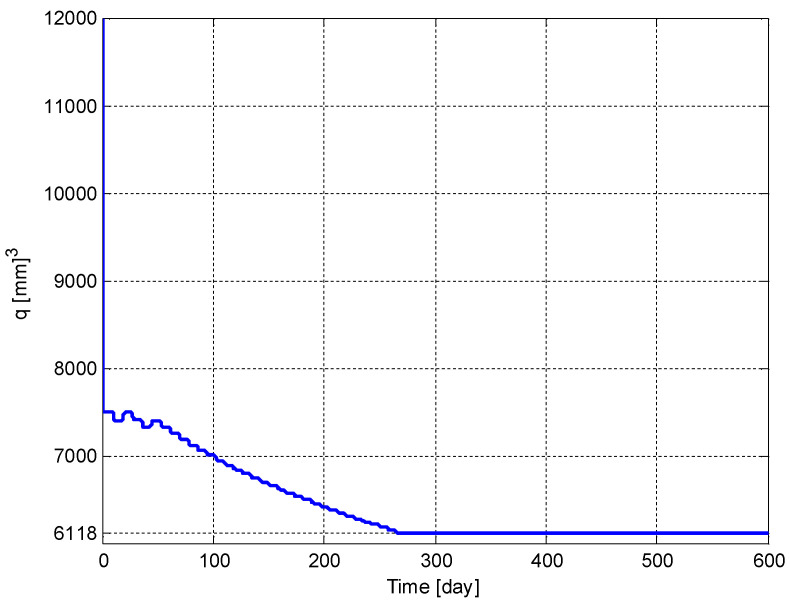
The time evolution of the tumor volume, *q*(*t*).

The time evolution of the tumor volume, p(t), and the carrying capacity of the endothelial cell volume, q(t), are shown in Figure 11 and Figure 12, respectively. It can be seen from these figures that p(t) and q(t) converge to their respective optimum values $p*=6118{\;[\mathrm{mm}]}^{3}$ and $q*=6118{\;[\mathrm{mm}]}^{3}$ given in (5), respectively. It should be noted that if the initial conditions, $p(t_{0})$ and $q(t_{0})$, were chosen to be smaller values, then the convergence would have been faster.

**Figure 13:**
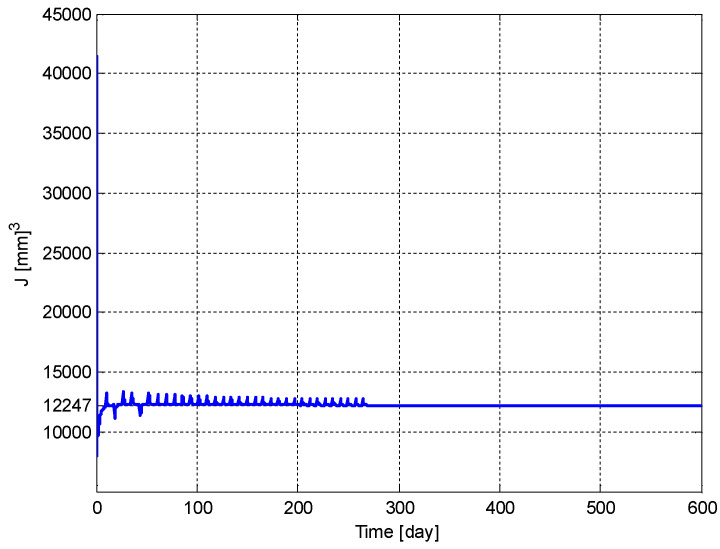
Time evolution of the estimate of the performance index, $\hat{J}(t)$.

The time evolution of the estimate of the performance index, $\hat{J}(t)$, is shown in Figure 13. It can be seen from Figure 13 that $\hat{J}(t)$ converges to $J*=12,247{\;[\mathrm{mm}]}^{3}$.

**Figure 14:**
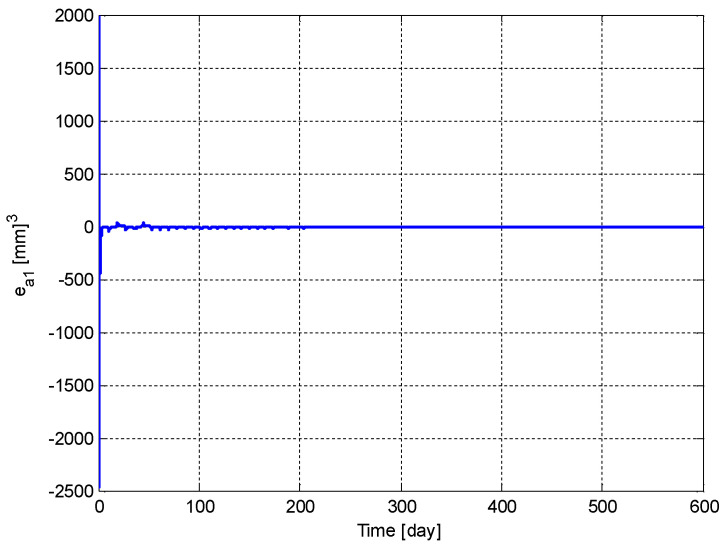
Variation of the tracking error, $e_{a1}(t)$.

Figure 14 shows the tracking error, $e_{a1}(t)$. It can be seen from the figure that the tracking error, $e_{a1}(t)$, is driven to zero.

Recently, an optimization method has also been proposed to analyze the mathematical model of cancer chemotherapy, which includes anti-angiogenic effects of cytotoxic agents in [37]. Similar to the study in [38], it provides a very useful discussion on modeling and optimization aspects of the problem, but they are open-loop controllers, i.e., both studies try to minimize to tumor volume by using the anti-angiogenic agents without feedback. Some useful methods with state feedback are given in [39], but they lack any optimization algorithm; on the other hand, lack of optimization in [39] leads to a very important advantage of continuous protocols. A very useful controller is given in [40], but it assumes all the system parameters are known. The main benefits of the controller given in existing papers are that it offers a control of the tumor volume and optimization algorithm to the drug dose. However, the parameter estimation scheme creates a disadvantage for the implementation of the control input signal, which is the concentration of the antiangiogenic agents. As seen from Figure 10, time variation of the drug dose has very high chattering. Even if this variation is continuous, a real-world clinical application will be discrete in two ways: drugs are not administrated continuously and the tumor volume is not measured continuously. Therefore, the chattering may affect the performance of the proposed tumor shrinking procedure, according to the selected drug-delivery frequency, especially in the first 6 months of treatment. Effects of continuous and discrete applications of the drug dosing controller has also been discussed in detail in [11].

## 5. Conclusions

A novel approach for the administration of anti-angiogenic tumor treatment by considering the nonlinear tumor dynamics and drug delivery pharmacokinetics and pharmacodynamics was presented. A performance index was formulated and then minimized to obtain an optimum tumor volume that would be realized; using an optimum drug dose and a nonlinear tracking controller was proposed to drive the system states to this optimum point. It was shown that if all the system parameters are known, the tumor volume can be driven to its optimum value exponentially fast.

The problem is more complicated if the tumor model parameters are unknown; it was shown that the performance index can be estimated, using a least-squares strategy, and the optimum tumor volume trajectory can be generated numerically. The proposed least-squares estimation scheme proves that the estimation errors can be driven to zero, provided that the persistency of excitation condition is satisfied. An adaptive tracking controller then drives the system to this optimum. The proposed adaptive scheme ensures that the tumor volume and the carrying capacity of the vascular network go to their desired values globally and asymptotically. Numerical simulation results were presented to show the performance and feasibility of the proposed estimation and control approaches. The simulations demonstrate that the developed estimation and control strategies successfully minimize the tumor volume, along with the carrying capacity of endothelial cells, with an optimum drug dose, despite of the lack of knowledge about system parameters. It can be concluded from the simulation study that the proposed tumor minimization technique can efficiently reduce the tumor volume with an optimum drug dose.

## Figures and Tables

**Table 1:** Parameter Values [31].

Parameter	Value	Unit
*α*	1.08	[day]^−1^
*d*	3.63 × 10^−4^	[day]^−1^ [mm]^−2^
*b*	0.243	[day]^−1^
*G*	1.3	[day]^−1^ [conc.]^−1^

## Data Availability

Information on numerical simulations can be provided upon request.

## Appendix A. Implementable Form of the Filtered Signal *Q_f_*(*t*)

In order to obtain an implementable form of (25), an auxiliary filter signal ζ(t)∈ℝ is designed as:

(A1) $$\dot{\zeta }≜-\beta \zeta -\beta^{2}q\;;\zeta \left(t_{0}\right)=-\beta q\left(t_{0}\right).$$

Furthermore, the filter signal $Q_{f}(t)$ is defined as:

(A2)
$$Q_{f}≜\zeta +\beta q.$$

After taking the time derivative of (A2), the following expression is obtained:

(A3) $${\dot{Q}}_{f}=-\beta \left(\zeta +\beta q\right)+\beta \dot{q}$$

where (A1) was utilized. After substituting (A2) into (A3), the following expression can be obtained:

(A4) $${\dot{Q}}_{f}≜-\beta Q_{f}+\beta Q$$

which is the same as (25). Thus, it is clear that (A2) and (A3) can be implemented to obtain $Q_{f}(t)$ without measuring $\dot{q}(t).$

## Appendix B. Proof Details for Theorem 2

Consider the following nonnegative Lyapunov function:

(A5)
$$V_{e}=\frac{1}{2}{\tilde{\theta }}_{e}^{T}\Gamma^{-1}{\tilde{\theta }}_{e}.$$

Taking its time derivative results in:

(A6) $${\dot{V}}_{e}=-{\tilde{\theta }}_{e}^{T}\Gamma^{-1}{\dot{\hat{\theta }}}_{e}+\frac{1}{2}{\tilde{\theta }}_{e}^{T}{\dot{\Gamma }}^{-1}{\tilde{\theta }}_{e}$$

where the time derivative of (32) was utilized. After substituting (34) and (35) into (A6), the following expression, the following is obtained:

(A7) $${\dot{V}}_{e}=-\varepsilon^{2}+\frac{1}{2}\varepsilon^{2}$$

where (33) was utilized. This expression can be further simplified as:

(A8)
$${\dot{V}}_{e}=-\frac{1}{2}\varepsilon^{2}.$$

After integrating (A8), the following expression is obtained:

(A9)
$$\frac{1}{2}\int_{t_{0}}^{\infty }\varepsilon^{2}\left(\tau \right)d\tau =V_{e}\left(t_{0}\right)-V_{e}\left(\infty \right)$$

From (A5) and (A8), it can be concluded that $V_{e}\left(t\right)\in L_{\infty };$ thus, ${\tilde{\theta }}_{e}\left(t\right)\in L_{\infty }.$ From (A9), it is clear that $\varepsilon \left(t\right)\in L_{\infty }\cap L_{2}.$ Since ${\tilde{\theta }}_{e}\left(t\right)\in L_{\infty },$ from (32), it follows that ${\hat{\theta }}_{e}\left(t\right)\in L_{\infty }.$ Assuming $p\left(t\right),\;q\left(t\right),\;u\left(t\right)\in L_{\infty }$ (see Proof 3), from (22), it follows that $W_{e}\left(t\right)\in L_{\infty },$ and from (21), it follows that $Q\left(t\right)\in L_{\infty }.$ Since $Q\left(t\right),\;W_{e}(t)\in L_{\infty },$ from Remark 2, it follows that $Q_{f}(t),\;{\dot{Q}}_{f}(t),\;W_{f}(t),\;{\dot{W}}_{f}(t)\in L_{\infty }.$ Since $W_{f}(t),\;\varepsilon (t)\in L_{\infty },$ and from Remark 3, $\Gamma (t)\in L_{\infty },$ therefore, from (34), it can be concluded that ${\dot{\hat{\theta }}}_{e}\left(t\right)\in L_{\infty };$ thus, the time derivative of (32) can be utilized to show that ${\dot{\tilde{\theta }}}_{e}\left(t\right)\in L_{\infty }.$ Since ${\dot{W}}_{f}(t),\;{\dot{\tilde{\theta }}}_{e}\left(t\right)\in L_{\infty },$ the time derivative of (33) can be utilized to show that $\dot{\varepsilon }\left(t\right)\in L_{\infty }.$ Since $\varepsilon \left(t\right)\in L_{\infty }\cap L_{2}$ and $\dot{\varepsilon }\left(t\right)\in L_{\infty },$ from Barbalat’s Lemma [41], it can be concluded that ε→0 as t→∞; thus, $\left\|W_{f}(t){\tilde{\theta }}_{e}(t)\right\|\to 0$ as t→∞. Upon the satisfaction of PE condition [36] given in (37), it can be further concluded that $\left\|{\tilde{\theta }}_{e}\right\|\to 0$ as t→∞.

## Appendix C. Proof Details for Theorem 3

Consider the following nonnegative Lyapunov function:

(A10)
$$V_{2}=\frac{1}{2}A_{1}e_{a1}^{2}+\frac{1}{2}A_{2}e_{a2}^{2}+\frac{1}{2}\gamma_{1}^{-1}{\tilde{\theta }}_{2}^{T}{\tilde{\theta }}_{2}+\frac{1}{2}\gamma_{2}^{-1}{\tilde{\theta }}_{3}^{T}{\tilde{\theta }}_{3}.$$

Taking its time derivative and then substituting the dynamics of *e_a1_* and *e_a2_*, given by (45) and (48), respectively, then the updates laws given in (50) yields:

(A11)
$$\begin{array}{cc} {\dot{V}}_{2}= & e_{a1}\left[-k_{a1}e_{a1}-e_{a2}q+W_{2}{\tilde{\theta }}_{2}\right]\\ & +e_{a2}\left[-k_{a2}e_{a2}+qe_{a1}+W_{3}{\tilde{\theta }}_{3}\right]\\ & -\gamma_{1}^{-1}{\tilde{\theta }}_{2}^{T}\left[\gamma_{1}W_{2}^{T}e_{a1}\right]-\gamma_{2}^{-1}{\tilde{\theta }}_{3}^{T}\left[\gamma_{2}W_{3}^{T}e_{a2}\right]\\ {\dot{V}}_{2}= & -k_{a1}e_{a1}^{2}-k_{a2}e_{a2}^{2}. \end{array}$$

From (A11), the following inequalities can be written:

(A12)
$${\dot{V}}_{2}\leq -k_{a1}e_{a1}^{2}$$

(A13)
$${\dot{V}}_{2}\leq -k_{a2}e_{a2}^{2}.$$

After integrating (A12) and (A13), the following inequalities will be obtained:

(A14)
$$k_{1}\int_{t_{0}}^{\infty }e_{a1}^{2}\left(\tau \right)d\tau \leq V_{2}\left(t_{0}\right)-V_{2}\left(\infty \right)$$

(A15)
$$k_{2}\int_{t_{0}}^{\infty }e_{a2}^{2}\left(\tau \right)d\tau \leq V_{2}\left(t_{0}\right)-V_{2}\left(\infty \right).$$

From (A10) and (A11), it can be concluded that $V_{2}\left(t\right)\in L_{\infty };$ hence, $e_{a1}\left(t\right),$ $e_{a2}\left(t\right),$ ${\tilde{\theta }}_{2}\left(t\right),$ ${\tilde{\theta }}_{3}\left(t\right)\in L_{\infty }.$ From (A14) and (A15), it is clear that $e_{a1}\left(t\right),e_{a2}\left(t\right)\in L_{2}.$ Since $q_{d}\left(t\right)$ is designed to be bounded, from (38), $q_{d}\left(t\right)\in L_{\infty };$ thus, from Remark 1, $p\left(t\right)\in L_{\infty }.$ The fact that $\theta_{2}$ and $\theta_{3}$ are constant vectors and ${\tilde{\theta }}_{2}\left(t\right),\;{\tilde{\theta }}_{3}\left(t\right)\in L_{\infty },$ from (44) and (49), it follows that ${\hat{\theta }}_{2}\left(t\right),\;{\hat{\theta }}_{3}\left(t\right)\in L_{\infty }.$ Since $p\left(t\right),\;q\left(t\right),\;q_{d}\left(t\right)\in L_{\infty },$ it is clear that $W_{2}\left(t\right)\in L_{\infty }.$ Further, since ${\hat{\theta }}_{2}\left(t\right)\in L_{\infty },$ therefore, from (43), $c_{d}\in L_{\infty };$ thus, from (39), $c\left(t\right)\in L_{\infty }.$ From (50), it follows that ${\dot{\hat{\theta }}}_{2}\left(t\right)\in L_{\infty }.$ Since $c_{d}\left(t\right)$ is a function of bounded and continuous signals, after taking its time derivative, it follows that ${\dot{c}}_{d}\left(t\right)\in L_{\infty };$ therefore $W_{3}\in L_{\infty }.$ Now it follows from (50) that ${\dot{\hat{\theta }}}_{3}\left(t\right)\in L_{\infty }.$ From (47), it follows that $u\left(t\right)\in L_{\infty }.$ Since $e_{a1}\left(t\right),\;e_{a2}\left(t\right)\in L_{\infty }\cap L_{2},$ and from (42) and (46) ${\dot{e}}_{a1}\left(t\right),\;{\dot{e}}_{a2}\left(t\right)\in L_{\infty },$ from Barbalat’s Lemma [41], it can be concluded that $e_{a1}\left(t\right),\;e_{a2}\left(t\right)\to 0$ as t→∞.
